# The pattern of tumor progression on first-line immune checkpoint inhibitor-based systemic therapy for Chinese advanced hepatocellular carcinoma –CLEAP 004 study

**DOI:** 10.3389/fimmu.2024.1310239

**Published:** 2024-04-22

**Authors:** Chao-Xu Yang, Yang-Xun Pan, Feng Ye, Xiao-Dong Zhu, Jun Xue, Xi Li, Zhen-Gang Yuan, Lan Zhang, Li Xu, Yong-Jun Chen, Nan-Ya Wang, Hui-Chuan Sun, Xiu-Feng Liu, China Liver Cancer Study Group Young Investigators (CLEAP)

**Affiliations:** ^1^ Medical Oncology, Nanjing Jinling Hospital, Nanjing, China; ^2^ Department of Liver Surgery, Sun Yat-Sen University Cancer Center, Sun Yat-Sen University, Guangzhou, China; ^3^ Department of General Surgery, Ruijin Hospital, Shanghai Jiao Tong University School of Medicine, Shanghai, China; ^4^ Department of Liver Surgery and Transplantation, Liver Cancer Institute and Zhongshan Hospital, Fudan University, Shanghai, China; ^5^ Cancer Center, Union Hospital, Tongji Medical College, Huazhong University of Science and Technology, Wuhan, Hubei, China; ^6^ Oncology Department, the First Affiliated Hospital of Jilin University, Changchun, Jilin, China; ^7^ Department of Oncology, Eastern Hepatobiliary Surgery Hospital, Second Military Medical University (Navy Medical University), Shanghai, China; ^8^ Liver Cancer Institute, Zhongshan Hospital, Fudan University & Key Laboratory of Carcinogenesis and Cancer Invasion, Ministry of Education, Shanghai, China

**Keywords:** hepatocellular carcinoma, progression pattern, immunotherapy, postprogression survival, prognostic model

## Abstract

**Background:**

For decades, stratification criteria for first-line clinical studies have been highly uniform. However, there is no principle or consensus for restratification after systemic treatment progression based on immune checkpoint inhibitors (ICIs). The aim of this study was to assess the patterns of disease progression in patients with advanced hepatocellular carcinoma (HCC) who are not eligible for surgical intervention, following the use of immune checkpoint inhibitors.

**Methods:**

This is a retrospective study that involved patients with inoperable China liver stage (CNLC) IIIa and/or IIIb. The patients were treated at eight centers across China between January 2017 and October 2022. All patients received at least two cycles of first-line treatment containing immune checkpoint inhibitors. The patterns of disease progression were assessed using RECIST criteria 1.1. Different progression modes have been identified based on the characteristics of imaging progress. The study’s main outcome measures were post-progression survival (PPS) and overall survival (OS). Survival curves were plotted using the Kaplan-Meier method to compare the difference among the four groups. Subgroup analysis was conducted to compare the efficacy of different immunotherapy combinations. Variations in the efficacy of immunotherapy have also been noted across patient groups exhibiting alpha-fetoprotein (AFP) levels equal to or exceeding 400ng/mL, in contrast to those with AFP levels below 400ng/mL.

**Results:**

The study has identified four distinct patterns of progress, namely p-IIb, p-IIIa, p-IIIb, and p-IIIc. Diverse patterns of progress demonstrate notable variations in both PPS and OS. The group p-IIb had the longest PPS of 12.7m (95% 9.3-16.1) and OS 19.6m (95% 15.6-23.5), the remaining groups exhibited p-IIIb at PPS 10.5 months (95%CI: 7.9-13.1) and OS 19.2 months (95%CI 15.1-23.3). Similarly, p-IIIc at PPS 5.7 months (95%CI: 4.2-7.2) and OS 11.0 months (95%CI 9.0-12.9), while p-IIIa at PPS 3.4 months (95%CI: 2.7-4.1) and OS 8.2 months (95%CI 6.8-9.5) were also seen. Additional stratified analysis was conducted and showed there were no differences of immunotherapy alone or in combination in OS (HR= 0.92, 95%CI: 0.59-1.43, P=0.68) and PPS (HR= 0.88, 95%CI: 0.57-1.36, P=0.54); there was no significant difference in PPS (HR=0.79, 95% CI: 0.55-1.12, P=0.15) and OS (HR=0.86, 95% CI: 0.61-1.24, P=0.39) for patients with AFP levels at or over 400ng/mL. However, it was observed that patients with AFP levels above 400ng/mL experienced a shorter median progression of PPS (8.0 months vs. 5.0 months) after undergoing immunotherapy.

**Conclusion:**

In this investigation of advanced hepatocellular carcinoma among Chinese patients treated with immune checkpoint inhibitors, we identified four distinct progression patterns (p-IIb, p-IIIa, p-IIIb and p-IIIc) that showed significant differences in PPS and OS. These findings demonstrate the heterogeneity of disease progression and prognosis after immunotherapy failure. Further validation in large cohorts is necessary to develop prognostic models that integrate distinct progression patterns to guide subsequent treatment decisions. Additionally, post-immunotherapy progression in patients with AFP levels ≥400ng/mL indicates a shortened median PPS. These findings provide valuable insights for future personalized treatment decisions.

## Introduction

Hepatocellular carcinoma (HCC) is responsible for the majority of primary liver cancers. Hepatocellular carcinoma is positioned as the third leading cause of cancer-related mortality on a global scale (1, 2). Certain people seek medical intervention at an advanced stage of incurable disease due to the concealment of HCC. Systemic therapy may represent the sole viable approach to enhance survival rates among patients diagnosed with advanced HCC or those who are deemed unsuitable candidates for significant surgical intervention or localized treatment, contingent upon the specific clinical stage of the patients (3). The landscape of systemic therapy for HCC has experienced significant changes following the global approval of immune checkpoint inhibitors (ICIs) as indications for systemic therapy towards the end of 2017 (4–13). Significantly, the Chinese National Health Commission Guidelines for liver Cancer, along with esteemed institutes such as NCCN, CSCO, AASLD, and EASL, continuously prioritize immunotherapy-based combination regimens as the primary approaches for the initial treatment phase of advanced HCC (14). Despite the considerable long-term life advantages offered by immunotherapy in the treatment of hepatocellular carcinoma (HCC), tumors do exhibit progression (9), commonly referred to as primary or acquired drug resistance (4, 7, 8, 15). The current diagnostic and risk stratification methods for predicting overall survival (OS) in the population following first immunotherapy are limited. Currently, the typical sequential patterns are ambiguous. This retrospective study aimed to analyze and summarize the imaging progression patterns of patients undergoing first-line immune or immune-combination therapy. The study aimed to establish the relationship between various progression patterns and overall survival (OS) and progression-free survival (PPS). The findings of this study offer a straightforward and comprehensible approach for determining patient prognosis.

## Materials and methods

### Patients

**Figure 1:**
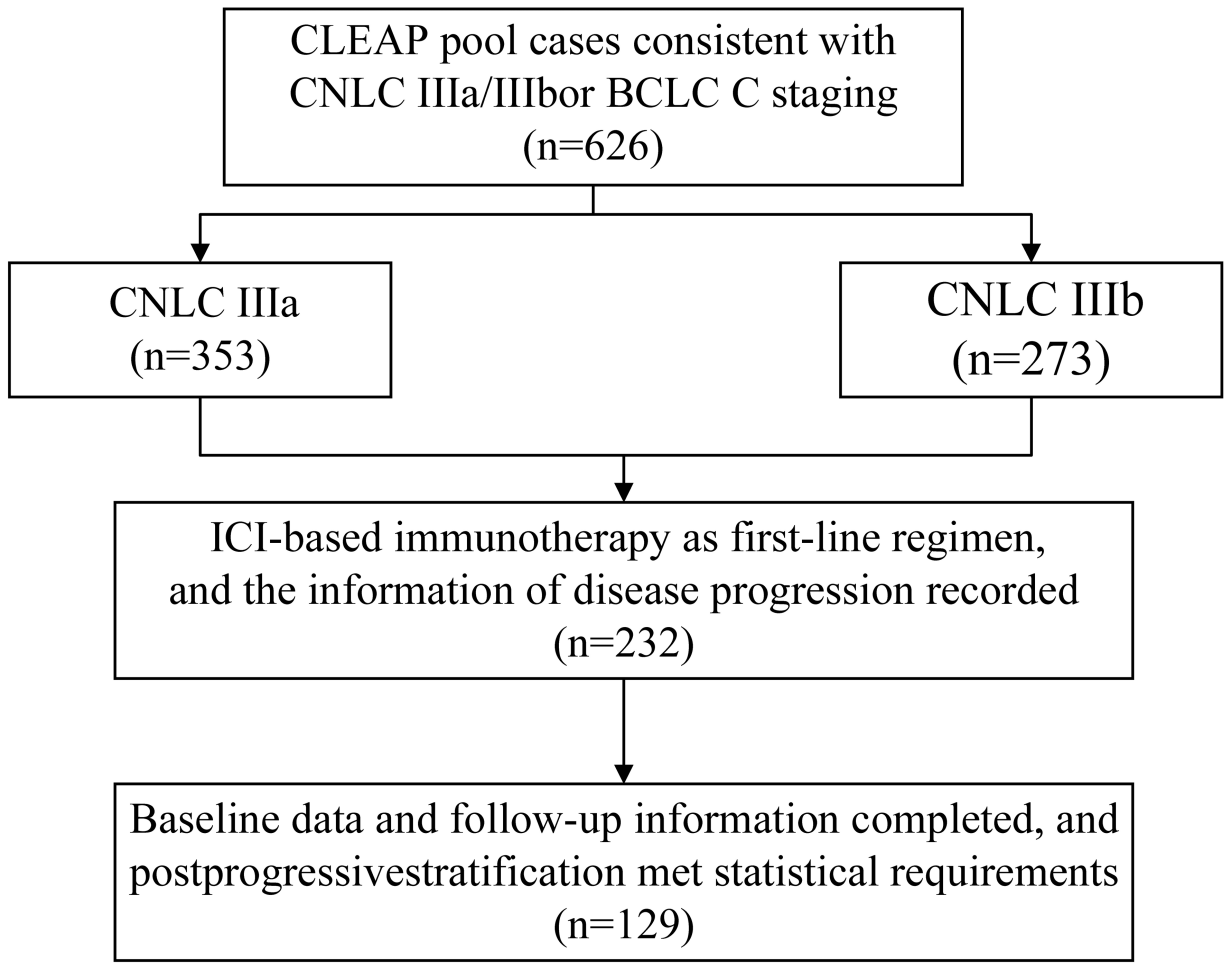
Patients disposition. CLEAP, China Liver Cancer Study Group Young Investigators; CNLC, China liver cancer.

The CLEAP database comprises information on 626 patients who were treated at eight clinical institutions in China between January 2017 and October 2022 for advanced HCC In order to be included in this study, patients must satisfy all of the following inclusion criteria: ①Minimum age ≥18 years old, ≤75 years old, regardless of gender; ② Hepatocellular carcinoma that has been diagnosed either histologically or clinically; ③ Not appropriate for surgical intervention, while having at least one detectable lesion (as per the RECIST version 1.1 criterion, which mandates a detectable lesion with a spiral CT scan length of at least 10mm or a malignant lymph node with a diameter of at least 15mm); ④ according to the Chinese Guidelines for the Diagnosis and Treatment of Primary Liver Cancer, the clinical diagnosis of hepatocellular carcinoma adheres to the established clinical diagnostic criteria. The present illness staging is classified as CNLC stage IIIa or IIIb, whereas BCLC is classified as Phase C, with a Child-Pugh class of B (CPB) or greater, and/or with an Eastern Cooperative Oncology Group performance status (ECOG PS) of 2 or greater; ⑤ Following at least one systematic therapy for hepatocellular carcinoma (HCC), the illness advances following first treatment. ⑥ The initial therapeutic approach should encompass the utilization of immune checkpoint inhibitors, namely PD-1 antibodies or PD-L1 antibodies. ⑦ The child’s Pugh score was found to be 7 points, indicating an A/B level, prior to the administration of second-line medication. ⑧ The clinical data is comprehensive and suitable for assessment and categorization. ⑨ Patients should have a minimum of one enhanced CT/MRI scan both before to and following therapy at their respective healthcare facilities in order to assess the effectiveness of the treatment. The criteria for exclusion were as follows: ① The patient’s overall health is poor and they are unable to tolerate systemic treatment. ② The patient has a pathological diagnosis of mixed hepatocellular carcinoma (HCC) along with intrahepatic cholangiocarcinoma or other non-HCC malignancies. ③ The patient has previously or concurrently had another malignancy. ④ The patient has undergone an organ transplant. ⑤ There are intrahepatic lesions that cannot be measured. The final number of patients enrolled was 129 (**Figure 1**). A retrospective analysis was conducted on the clinical records of the cohort that was first recruited in ICIs therapy. The clinical data collected during the diagnosis of hepatocellular carcinoma (HCC) in cirrhotic patients was assessed using histological confirmation or non-invasive imaging criteria.The baseline characteristics of the enrolled patients were seen in **Table 1**.

**Table 1:** Baseline characteristics of the clusters included in the analysis.

	p-IIb (n=35)	p-IIIa (n=30)	p-IIIb (n=30)	p-IIIc (n=34)
Age (year)				
	51.6±8.3	53.5±10.8	48.6±11.6	56.5±8.8
Gender				
female	2	6	4	5
male	33	24	26	29
BCLC				
B	1	0	0	1
C	34	30	30	33
Child Pugh				
A	34	23	21	34
B	1	7	9	0
C	0	0	1	0
AFP				
<400ng/mL	15	10	10	17
≥400ng/mL	20	20	20	17
IO				
Monotherapy	7	7	3	10
Combination therapy	28	23	27	24

## Methods

### Progression patterns defined

**Figure 2:**
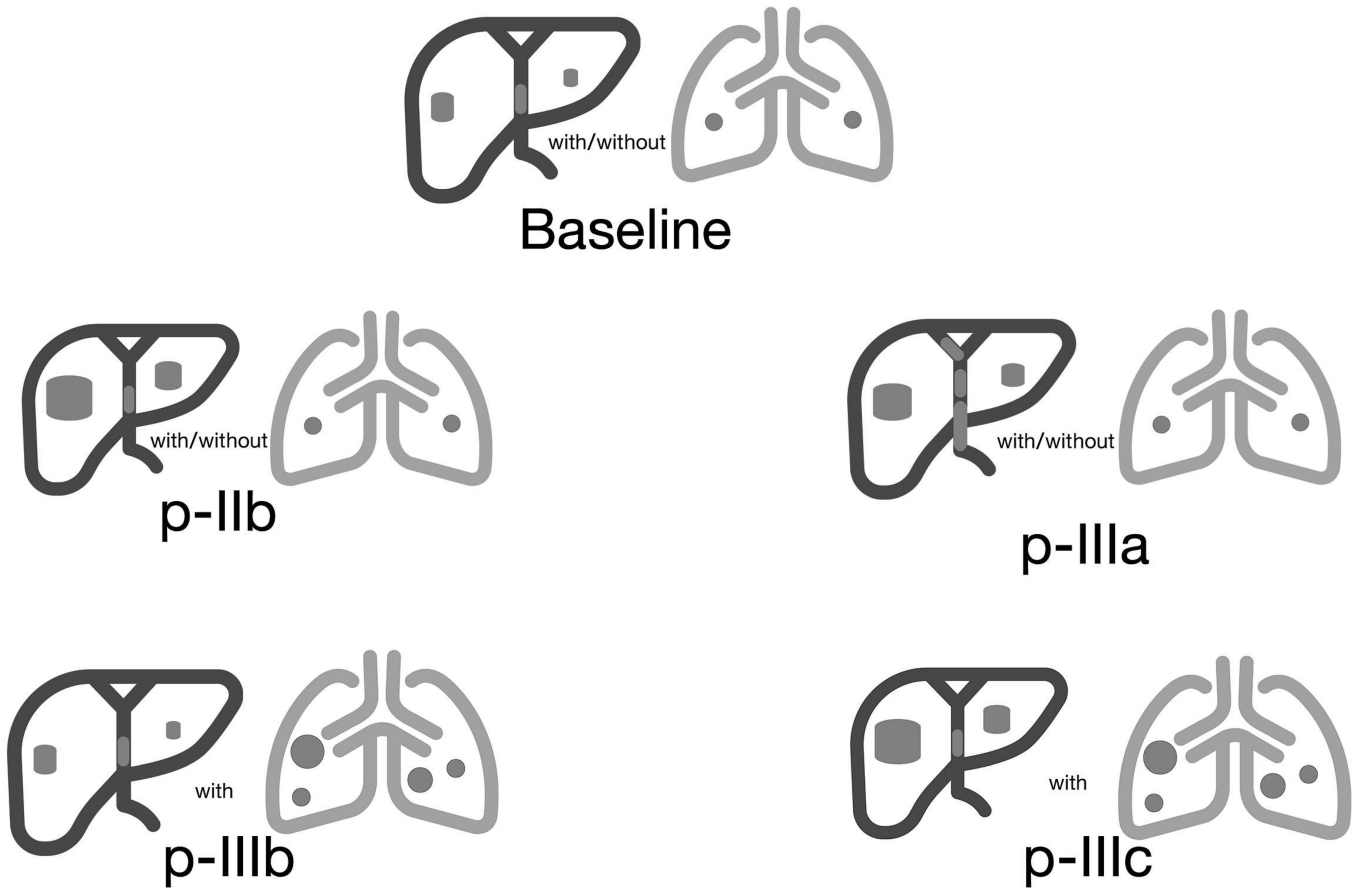
Four patterns of progression.

In accordance with the initial disease stage and the manner of disease advancement, and considering the unique context of China, based on the manner in which diseases progress, we have refined the established clusters initially formulated by Jordi Bruix in 2019 (16). specifically, the progression mode was divided into four distinct groups, as depicted in **Figure 2**. ① A post-progression phase IIb (p-IIb) is characterized by a size expansion of the target liver lesion over 20% and/or the emergence of new lesions in the liver without any invasion of intrahepatic blood vessels. ②Compared to the baseline period, the liver shows new lesions or an increase of over 20% in the original lesions, along with new intrahepatic vascular invasion or stable liver lesions, with the appearance of new intrahepatic vascular invasion. (designated as post-progression phase IIIa, p-IIIa). ③ The progression of extrahepatic lesions occurs while intrahepatic lesions stay constant, which is commonly referred to as stage IIIb progression (p-IIIb). ④The simultaneous occurrence of two or more types of progression described in points 1, 2, and 3, referred to as extensive progression of both intrahepatic and extrahepatic lesions (referred to as post-progression phase IIIc, p-IIIc).

The major outcomes, namely PPS and OS, were compared across the various groups. A total of 129 patients who met the eligibility criteria and had comprehensive baseline and follow-up data, and who had undergone a minimum of 2 cycles of Immunotherapy as their initial treatment, were included in the study. A minimum of two cycles of immunotherapy (PD-1/PD-L1) were administered to the patients, either with or without small molecule tyrosine-targeted medicines (TKIs), as well as bevacizumab or becacizumab biosimilars. The disease development patterns were evaluated using the Immune RECIST 1.1 criteria. The database records the most recent date of initial therapy as August 2022. It is important to mention that the patients in the study had a liver function that was classified as Child Pugh class A or, at most, B7 when they started their first-line medication. This research requires the categorization of disease progression patterns and the ongoing monitoring of all participants involved in the study until their demise. In addition, a subgroup analysis was conducted on persons who received different combinations of medication and had different levels of alpha-fetoprotein (AFP).

### Statistical analyses

The Kaplan-Meier technique was employed to compute the time intervals, which were subsequently compared across several groups using the log-rank test.To delve deeper into the analysis, we conducted a meticulous stratified examination, taking into account the administration of immune checkpoint inhibitor (ICI) monotherapy or combination therapy, as well as the AFP level (measured in ng/mL). The statistical analyses were executed utilizing the esteemed software packages SPSS 26.0 and Graph Pad Prism 9 and. Rsoftware version 4.3.3 (http://www.r-project.org/). P-values of <0.05 were considered statistically significant.

## Results

**Figure 3:**
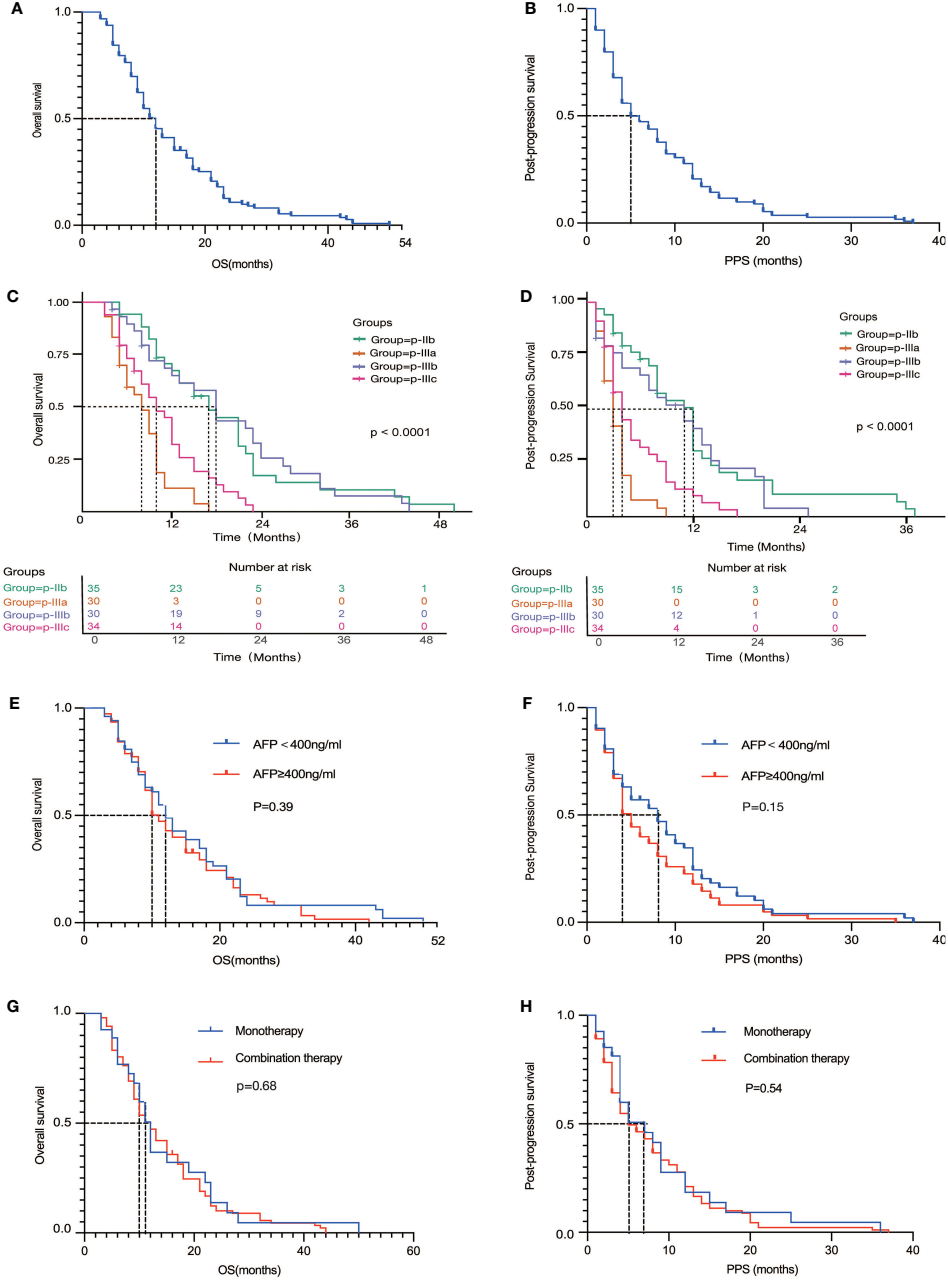
**(A, B)** Overall survival **(A)** and Progression-free survival **(B)** for the entire cohort of patients; **(C, D)** Overall survival **(C)** and Progression-free survival **(D)** of four different progression patterns; **(E, F)** Overall survival **(E)** and Progression-free survival **(F)** of different AFP levels; **(G, H)** Overall survival **(G)** and Progression-free survival **(H)** of monotherapy and combination therapy.

In this retrospective study involving 129 Chinese patients with advanced hepatocellular carcinoma (HCC) treated with immune checkpoint inhibitors (ICIs), **Figures 3A, B** showed the| PPS and overall survival (OS) of all eligible patients (N = 129). we identified four distinct patterns of disease progression: p-IIb, p-IIIa, p-IIIb, and p-IIIc. (**Figure 2**) These patterns underscore the heterogeneity in HCC’s response to immunotherapy and its impact on patient outcomes, particularly post-progression survival (PPS) and overall survival (OS) (**Figures 3C, D**).

The p-IIb pattern, characterized by the expansion of target liver lesions by more than 20% and/or the appearance of new lesions without intrahepatic vascular invasion, was associated with the longest PPS of 12.7 months (95% CI: 9.3-16.1) and OS of 19.6 months (95% CI: 15.6-23.5), suggesting a relatively high degree of immune control over tumor growth. Conversely, the p-IIIa pattern, indicative of a more aggressive disease behavior with new intrahepatic lesions or vascular invasion, resulted in a significantly shorter PPS of 3.4 months (95% CI: 2.7-4.1) and OS of 8.2 months (95% CI: 6.8-9.5). Patients exhibiting the p-IIIb pattern, with progression of extrahepatic lesions, and those with the p-IIIc pattern, representing extensive progression involving both intrahepatic and extrahepatic disease, highlighted the capability of HCC to metastasize and evade immune surveillance, with corresponding impacts on survival rates.

Subgroup analysis further elucidated the complex relationship between treatment efficacy and tumor biology. No statistically significant difference was found in the efficacy of ICIs, whether used as monotherapy or in combination in OS (HR= 0.92, 95%CI: 0.59-1.43, P=0.68) and PPS (HR= 0.88, 95%CI: 0.57-1.36, P=0.54), emphasizing the need for a deeper understanding of the factors influencing treatment response. The effects of immunotherapy, both as monotherapy and in combination with other agents, did not demonstrate statistically significant differences across the different progression patterns. This suggests a complex interaction between tumor biology and treatment efficacy that transcends simple therapeutic categorization (17).

In addition, the predictive usefulness of AFP levels, a biomarker that has traditionally been linked to the prognosis of hepatocellular carcinoma (HCC), was examined following immunotherapy. Nevertheless, there was no notable disparity in patients with AFP levels lower or greater than 400ng/mL in terms of PPS (HR=0.79, 95% CI: 0.55-1.12, P=0.15) and OS (HR=0.86, 95% CI: 0.61-1.24, P=0.39). The analysis of the AFP Subgroup yielded detailed insights into the effectiveness of ICIs in this specific group of patients. It is noteworthy that individuals who had AFP levels equal to or over 400ng/mL had a significant reduction in median PPS from 8.0 months to 5.0 months, in contrast to those with lower AFP levels. The differential analysis highlights the predictive importance of AFP in the context of immunotherapy, suggesting its potential usefulness in customizing treatment methods for individual patients.

To augment the textual description of our results, several Kaplan-Meier survival curves were plotted, illustrating the survival disparities among the four progression patterns. These visual representations underscore the prognostic heterogeneity inherent in HCC progression post-ICI treatment and highlight the critical need for personalized therapeutic approaches.

The findings from this study not only reveal the distinct progression patterns in advanced HCC following ICI treatment but also highlight the prognostic heterogeneity and the importance of considering factors such as AFP levels in post-progression therapeutic strategies. The integration of these insights into clinical practice could facilitate the development of more personalized treatment approaches, ultimately improving patient outcomes in this challenging context.

## Discussion

This study systematically analyzed the patterns of disease progression in Chinese patients with advanced hepatocellular carcinoma (HCC) treated with immune checkpoint inhibitors (ICIs), uncovering four distinct progression patterns (p-IIb, p-IIIa, p-IIIb, and p-IIIc) associated with significant differences in post-progression survival (PPS) and overall survival (OS) (18–23). These findings not only highlight the heterogeneity of HCC progression following ICI therapy but also suggest the potential for these patterns to serve as prognostic indicators for tailoring subsequent treatment strategies.

The observed variability in survival outcomes across the different progression patterns underscores the complexity of HCC response to immunotherapy. The relatively longer PPS and OS in patients with the p-IIb pattern suggest that certain biological behaviors of HCC, such as the lack of intrahepatic vascular invasion and limited disease progression, may be more amenable to subsequent therapeutic interventions. In contrast, patterns characterized by more aggressive disease progression, such as p-IIIa and p-IIIc, were associated with poorer survival outcomes, indicating a potential need for more aggressive or alternative treatment approaches.

Our findings also draw attention to the role of alpha-fetoprotein (AFP) levels in predicting treatment outcomes. Patients with AFP levels ≥400ng/mL experienced a shortened median PPS and OS post-immunotherapy, aligning with previous studies that have identified high AFP levels as a marker of poor prognosis in HCC[^4]. This observation reinforces the importance of considering AFP levels in the decision-making process for post-progression treatment strategies.

The lack of significant differences in survival outcomes between patients treated with immunotherapy alone versus in combination suggests that the benefit of combination therapies may not be universally applicable and highlights the need for personalized treatment approaches based on individual patient characteristics and disease progression patterns (17, 24).

It is important to note the limitations of our study, including its retrospective nature and the potential for selection bias due to the study’s inclusion criteria. Additionally, the heterogeneity of the patient population and the treatment regimens used across the participating centers may have influenced the observed outcomes.

In conclusion, our study provides valuable insights into the patterns of disease progression in HCC patients treated with ICIs and underscores the complexity of predicting and managing post-immunotherapy progression. These findings advocate for further validation in larger cohorts and the development of comprehensive prognostic models that integrate distinct progression patterns and biomarkers such as AFP levels. Ultimately, such models could guide clinicians in optimizing post-progression treatment strategies, thereby improving patient outcomes in advanced HCC.

## Data availability statement

The original contributions presented in the study are included in the article/supplementary material. Further inquiries can be directed to the corresponding authors.

## Ethics statement

The studies involving humans were approved by Ethics Committee of Zhongshan Hospital Affiliated to Fudan University. The studies were conducted in accordance with the local legislation and institutional requirements. Written informed consent for participation was not required from the participants or the participants’ legal guardians/next of kin in accordance with the national legislation and institutional requirements.

## Author contributions

CY: Investigation, Writing – original draft, Validation, Writing – review & editing. YP: Data curation, Investigation, Writing – review & editing. FY: Data curation, Writing – review & editing. XZ: Conceptualization, Writing – original draft. JX: Writing – original draft, Conceptualization, Investigation. XiL: Writing – original draft, Conceptualization, Investigation. ZY: Writing – original draft, Conceptualization, Investigation. LZ: Writing – original draft, Conceptualization, Investigation. LX: Conceptualization, Investigation, Writing – original draft. YC: Conceptualization, Writing – original draft. NW: Conceptualization, Writing – original draft. HS: Writing – original draft, Writing – review & editing. XiuL: Writing – original draft, Writing – review & editing.
